# Quantification of late gadolinium enhanced CMR in viability assessment in chronic ischemic heart disease: a comparison to functional outcome

**DOI:** 10.1186/1532-429X-11-6

**Published:** 2009-03-09

**Authors:** Aernout M Beek, Olga Bondarenko, Farshid Afsharzada, Albert C van Rossum

**Affiliations:** 1 Department of Cardiology, VU University Medical Center, Amsterdam, The Netherlands; 2 Interuniversity Cardiology Institute of the Netherlands, Utrecht, The Netherlands

## Abstract

**Background:**

Quantification of late gadolinium enhanced cardiovascular magnetic resonance (LGE CMR) by objective window setting increases reproducibility and facilitates multicenter comparison and cooperation. So far, quantification methods or models have only been validated to postmortem animal studies. This study was undertaken to evaluate quantification of LGE in relation to the clinical standard of viability, i.e. functional outcome after revascularization.

Thirty-eight patients with chronic ischemic myocardial dysfunction underwent cine and LGE 1 month before and cine CMR 6 months after coronary revascularization. Enhancement was quantified by thresholding window setting at: 2-8SD above mean signal intensity of a remote normal region, and according to the full width at half maximum method (FWHM). Dysfunctional segments were divided in 5 groups according to segmental extent of enhancement (SEE): SEE 1 – no enhancement to SEE 5 – 76–100% with each quantification method.

**Results:**

Quantification methods had a strong influence on SEE and total infarct size. Multilevel analysis showed that thresholding contrast images at 6SD best predicted segmental functional outcome after revascularization, but the difference with other methods was small and non-significant.

**Conclusion:**

Simple thresholding techniques strongly influence global and segmental extent of LGE, but have relatively little influence on the accuracy to predict segmental functional improvement after revascularization.

## Background

Revascularization of dysfunctional but viable myocardium may lead to reversed remodelling, improved regional and global function and better prognosis in patients with chronic ischemic heart disease [1]. The diagnostic accuracy of imaging modalities to predict functional outcome is influenced by the definition of disease (what is viable). Although visual or qualitative analysis may provide satisfactory results, standardization and quantification of these definitions increases reproducibility and reliability in follow-up studies, and facilitates comparison between different centers.

Late gadolinium enhanced cardiovascular magnetic resonance (LGE CMR) accurately visualizes the transmural extent of ischemia-related scar and has been shown to predict the likelihood of functional improvement after revascularization [2,3]. Several methods have been proposed to differentiate enhanced, non-viable from non-enhancing, viable myocardium, all using the in-slice signal intensity of infarcted or remote myocardium, and ranging from simple thresholding to more complex computer algorithms. We have previously shown that the use of common thresholds based on the suppressed signal of remote myocardium may lead to considerable overestimation of the infarct size [4]. However, in this study, we used visual estimation as the reference standard. Although a number of experimental studies have used ex-vivo imaging or 2,3,5-triphenyltetrazolium choride (TTC) staining to determine the optimal threshold of enhancement, so far, no study has used the clinically useful standard of viability i.e. functional outcome after revascularization [5-7].

Therefore, the aim of this study was to evaluate the relation between quantification of LGE and functional outcome after revascularization in patients with chronic ischemic myocardial dysfunction. To quantify LGE, we chose simple thresholding techniques that are easily applicable in any clinical or research situation.

## Methods

### Patients

All patients with known coronary artery disease and regional wall motion abnormalities on echocardiography or left ventricular (LV) angiography, without CMR contraindications, who were scheduled to undergo surgical or percutaneous revascularisation, were study candidates. The Committee on Research Involving Human Subjects of the VU University Medical Centre, Amsterdam, approved the study protocol. All patients gave written informed consent.

Forty-seven patients were initially included in this study protocol. After revascularization, 7 patients were excluded because of left ventricular aneurysmectomy (1), electrocardiographic and/or biochemical evidence of peri-procedural myocardial infarction (defined as post procedural peak CK-MB > 3 upper limit of normal) (4), pacemaker implantation (1), and incomplete data (1). During analysis, 2 more patients were excluded because of absence of wall motion abnormalities at baseline and non-diagnostic image quality, leaving 38 patients as the final study group. All patients were in stable clinical condition at the time of both CMR examinations without clinical evidence of ischemic events during the study period.

### CMR

CMR scans were acquired at 4 ± 4 weeks before and 30 ± 4 weeks after revascularisation. All scans were performed on a 1.5T scanner (Sonata, Siemens, Erlangen, Germany) with the patient in a supine position using a four-element phased array cardiac receiver coil. ECG-gated cine images were acquired using a breath-hold segmented steady-state free precession sequence (true FISP; echo time/repetition time of 1.2/3.2 ms; resolution of 1.3 × 1.8 × 5 mm). Per patient eight to ten short-axis views were obtained every 10 mm starting from the mitral valve insertion and covering the entire left ventricle. Ten to 15 minutes after injection of a gadolinium-based contrast agent (Magnevist, Schering AG, Berlin, Germany; 0,2 mmol/kg) contrast-enhanced images were acquired in the same orientation as the cine images using a 2D-segmented inversion recovery gradient-echo pulse sequence triggered to end-diastole (repetition time/echo time = 9.6/4.4 ms, flip angle 25°, inversion time set to suppress signal from remote myocardium, matrix 208 × 256 and a typical voxel size of 1.6 × 1.3 × 5.0).

### Data analysis

All data were analysed on a separate workstation using a dedicated software package (MASS v15, 2008, Medis, Leiden, The Netherlands).

#### Segmental function

Segmental wall thickness was measured at end-systole and end-diastole after manual tracing of endocardial end epicardial borders in stop-frame images, carefully excluding trabeculations and papillary muscles. The observer (OB) was blinded to other patient or imaging data such as extent of coronary artery disease, use of medication and results of the LGE analysis. Baseline and follow-up slices were analysed separately after registration using the scanner slice position and various anatomical landmarks such as right ventricle septal insertion sites, papillary muscle location, and trabecularization patterns in the right and left ventricles. Although the observer was unaware of the timing of the study, blinding to pre-/postoperative status was impossible because of the artifacts related to sternal wires. For segmental analysis, the 2 most basal and apical slices were excluded because of the left ventricular outflow tract and partial volume effects, respectively. The remaining slices were divided into 6 segments each, starting at the inferior insertion of the right ventricle to the septum.

Segmental wall thickening (SWT) in millimetres was calculated as: end systolic wall thickness minus end diastolic wall thickness. Segments with SWT < 3 mm (mean – 2SD) were considered dysfunctional [4]. Functional improvement was defined as an increase in SWT of ≥ 1.5 mm compared to baseline. Intraobserver and interobserver variability of SWT were 0.0 ± 0.4 mm (mean difference between 2 measurements (OB); intraclass correlation coefficient = 0.97, 95% confidence interval 0.86 – 0.99), and 0.1 ± 0.7 mm (mean difference between values of observer 1 (OB) and 2 (AMB); intraclass correlation coefficient = 0.89, 95% confidence interval 0.55–0.98), as reported previously [3].

#### Global function

Left ventricular end-diastolic volume (EDV) and end-systolic volumes (ESV) were determined by planimetry of all short-axis images in each patient and indexed to body surface area. Left ventricular ejection fraction (EF, %) was calculated as (LVEDV - LVESV)/LVEDV * 100%.

#### Enhancement

Endocardial and epicardial contours were manually traced, again avoiding papillary muscles and trabecularizations. Enhanced regions were then determined in the following ways:

I. After thresholding signal intensity at 2–8 standard deviations (SD) above the mean signal intensity of remote normal myocardium in the same slice. A region of interest of 0.5–1 cm^2 ^was manually drawn and placed in remote normal myocardium (defined as normal function without enhancement on visual assessment). If a slice contained no remote normal myocardium, mean and SD of the nearest slice with normal remote myocardium was used; if the 2 neighbouring slices contained normal myocardium, mean and SD were averaged.

II. After thresholding signal intensity using the full-width at half-maximum (FWHM) method that defines the enhanced area by using 50% of the maximum signal found within the enhanced area. The maximum signal was found by computer-assisted window thresholding of the enhanced area. If no enhancement was found in a slice, the maximum signal of the nearest slice with enhancement was used; if the 2 neighbouring slices showed enhancement, maximum signals were averaged.

All areas of enhancement were quantified by computer-assisted planimetry on each of the short axis images and segmental extent of enhancement (SEE) was expressed as percentage of segmental area. Obvious artifacts such as caused by motion were excluded by highlighting them using a tool from the software package. Other small isolated regions of enhancement that were clearly not of ischemic origin (like small subepicardial spots) were also excluded from analysis. Total infarct size was calculated by summation of all slice volumes of enhancement, i.e. including the slices that were excluded for segmental analysis.

### Statistical analysis

The paired sample *t *test (with Bonferroni correction) and the independent samples *t *test were used to compare means within the study group or between subgroups. To account for the non-independence of the data, we used multilevel logistic regression (MlwiN, version 1.02.0007, Centre for Multilevel Modelling, London, United Kingdom) to analyse the relation between SEE and the likelihood of improvement. Details of the multilevel analysis have been published previously [3]. All dysfunctional segments were assigned to one of the following groups according to SEE: 1 – 0%, 2 – 1 to 25%, 3 – 26 to 50%, 4 – 51 to 75%, and 5 – 76 to 100% enhancement. The regression coefficients were used to calculate odds ratios that express the likelihood of improvement of SEE-groups 2–5 relative to SEE-group 1. To compare the different quantification methods, multilevel analysis was repeated assuming a linear correlation between SEE and likelihood of improvement, which generates one regression coefficient per method.

Receiver operating characteristics (ROC) analysis was used to find the SEE cut-off with highest diagnostic accuracy to predict segmental functional improvement.

Improvement in ejection fraction was defined as an increase from baseline to follow-up of ≥ 5%. Since the number of viable segments per patient is not normally distributed, the Mann-Whitney test was used to compare these between patients with and without improved ejection fraction.

All values are expressed as mean ± SD. P-values < 0.05 were considered statistically significant.

## Results

Patient characteristics are provided in table 1.

**Table 1:** Patient characteristics

Number of patients	38
Age (sd)	62 (10)

Men	33 (87%)

Diabetes	10 (26%)

Hypercholesterolemia	7 (18%)

Hypertension	8 (21%)

Smoking	4 (11%)

1-vessel disease	10 (26%)

2-vessel disease	24 (63%)

Medication	
*Aspirin*	22 (58%)
*Acenocoumarol*	16 (42%)
*Betablockers*	29 (76%)
*Statins*	29 (76%)
*Ace-inhibitors*	20 (53%)

PCI*	9 (24%)

CABG*	30 (76%)
*Mean nr grafts (sd)*	3,6 (1,1)

* percutaneous coronary intervention.

** coronary artery bypass grafting.

### Extent of enhancement

The extent of enhancement according to quantification method is shown in table 2. Both total infarct size and segmental extent of enhancement were strongly correlated between all methods (p < 0.001), except total infarct size between FWHM and 2SD and 3SD (non-significant after Bonferroni correction). Total infarct size decreased with increasing number of SD's (all steps p < 0.001). Total infarct size according to FWHM differed significantly from 2–4 SD (p < 0.001). Mean SEE decreased with increasing number of SD's and FWHM (all p < 0.001).

**Table 2:** Global and segmental extent of enhancement according to quantification

	2SD	3SD	4SD	5SD	6SD	7SD	8SD	FWHM
TIS	31.3 (12.1)	23.6 (11.6)	18.7 (11.5)	15.7 (11.1)	12.6 (9.1)	10.4 (8.9)	7.9 (6.8)	14.1 (6.8)

SEE*	36.2 (31.9)	29.2 (31.0)	24.3 (29.5)	20.9 (27.9)	17.9 (25.8)	15.8 (24.4)	14.1 (23.3)	15.4 (22.4)

TIS = mean (sd) total infarct size. SEE = mean (sd) segmental extent of enhancement.

* = dysfunctional segments only. Statistical analysis see text.

### Functional improvement

A total of 1122 segments were analysed (29.6 ± 5.5/patient). Of these, 628 were dysfunctional (mean SWT 1.1 ± 1.1 mm). Segmental function improved in 174 segments (mean SWT 0.9 ± 1.2 to 3.5 ± 1.5 mm). The likelihood of improvement was inversely related to the SEE according to all quantification methods. The results of the multilevel analysis are displayed in table 3. The odds ratios were inverted so that they reflect the number of times that a certain SEE group is less likely to improve compared to SEE group 1 in that category. Quantifying enhancement using 6SD best predicted segmental functional improvement at follow-up: segments in SEE-groups 2, 3, 4 and 5 were 2.7, 4.7, 5.2 and 14.8 less likely to improve than segments in SEE-group 1 (table 3).

**Table 3:** Likelihood of improvement versus quantification.

	SEE 1	SEE 2	SEE 3	SEE 4	SEE 5
2SD	1	0,7	1,2	2,0	2,5

3SD	1	1,6	3,8	3,8	5,2

4SD	1	1,3	1,5	3,4	4,5

5SD	1	1,5	3,1	4,3	6,1

6SD	1	2,9	4,7	5,2	14,8

7SD	1	2,0	4,4	3,9	10,8

8SD	1	2,1	4,1	3,2	9,6

FWHM	1	2,4	3,5	4,7	5,5

SEE = segmental extent of hyperenhancement. Numbers represent odds ratios of likelihood of improvement of SEE 2–5 versus SEE 1, which is set at 1.

**Figure 1:**
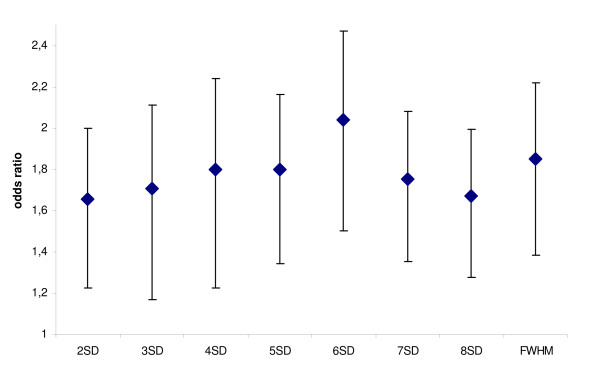
**Multilevel analysis assuming linear relation between likelihood of improvement according and quantification method**. (Inverted) odds ratios and 95%-confidence intervals according to quantification method. See text for further explanation.

Multilevel analysis was repeated assuming a linear relationship between SEE-groups and likelihood of improvement. This analysis generates one odds ratio that represents the likelihood of improvement from one SEE-group to the next. 6SD had the highest predictive power: SEE-group x was 2.04 (95% confidence intervals 1.62–2.58) less likely to improve than the SEE-group x-1. 2SD had the lowest power with (inverted) odds ratio 1.66 (1.31–2.08). Figure 1 shows 95%-confidence intervals for each method, demonstrating that there is considerable overlap.

Left ventricular EDV and ESV were 123 ± 36 ml/m2 and 79 ± 35 ml/m2 at baseline, and decreased significantly to 108 ± 36 ml/m2 (p < 0.001) and 68 ± 35 ml/m2 (p = 0.002), respectively. Mean EF improved from 38.2 ± 11.4% at baseline to 40.4 ± 12% at follow-up (p = 0.067).

ROC analysis showed that the 6SD method optimally predicted segmental improvement at < 10% SEE (AUC 0.700 (0.654–0.746), sensitivity 70%, specificity 65%) (table 4). Twenty-two patients showed significant (≥ 5%) improvement in EF. Although the number of dysfunctional segments with < 10% SEE-6SD was higher in patients with improved ejection fraction compared to patients without improvement (median 7.5, range 1–24, vs 4.0, range 0–14), there was considerable overlap, and no significant difference could be found (Mann-Whitney-test, p = 0.251).

**Table 4:** Sensitivity and specificity to quantification method.

	sens	spec	SEE*
2SD	0,72	0,55	28

3SD	0,72	0,57	21

4SD	0,64	0,65	25

5SD	0,68	0,64	14

6SD	0,70	0,65	9

7SD	0,65	1,00	9

8SD	0,65	1,00	7

FWHM	0,61	0,66	9

* = segmental extent of enhancement (%) at optimal sensitivity and specificity.

## Discussion

Our results show that quantification methods have a strong influence on total and regional infarct extent. Although analysis using 6SD above signal intensity of a remote normal region resulted in the best prediction of segmental functional improvement after revascularization, differences with the other methods were small and non-significant.

### LGE quantification

Both SD- and FWHM-methods are based on experience rather than on firm pathophysiological evidence. SD uses the (purposely suppressed) signal of a remote normal region, whereas FWHM uses the peak signal within the enhanced region. FWHM has been suggested on theoretical grounds to be less sensitive to partial volume effects. Amado et al compared total infarct size according to the SD- and FWHM-thresholding methods and postmortem data in an animal occlusion-reperfusion study [5]. They found that FWHM correlated best with TTC-staining with a small overestimation (significance not reported), whereas no significant correlation and considerable overestimation was found with 6SD. Heiberg et al recently proposed a quantification method to compensate for partial volume effects in thicker (8–10) mm) slices by weighting pixels according to their signal intensity [7]. The standard LGE protocol in our institution uses 5 mm slice thickness, which maintains excellent image quality while minimizing partial volume effects.

Hsu et al developed a computer algorithm based on feature analysis and combined thresholding involving sequential use of the 2SD- and FWHM-method [6]. The algorithm was validated in a canine study, and compared to visual assessment and 2SD- and FWHM-thresholding using ex-vivo and in-vivo LGE. The authors found that infarct size measured by their model showed no difference with TTC-staining, and was more accurate than the other methods, that all overestimated the histopathological infarct size. The algorithm was subsequently tested in 20 patients with acute or chronic infarction, and resulted in significantly lower values for both regional and global infarct size when compared to visual assessment, although this study lacked a reference standard [9]. Hsu et al used phase-sensitive reconstruction in both studies and also used 8 mm slice thickness, which makes comparison of their findings to our study difficult and again stresses the importance of standardization.

In a previous study we quantified enhancement in 15 patients with chronic ischemic heart disease. In comparison to visual assessment, we found significant overestimation with 2–4SD of both segmental extent and total infarct size [4]. The use of 6SD led to a significant underestimation, whereas 5SD showed no difference.

In the current study we used functional outcome after revascularisation as the clinical reference standard of viability. We found that, although the extent of enhancement was profoundly influenced by the quantification method used, there was no significant difference in their accuracy to predict segmental functional improvement at follow-up. 6SD showed the highest accuracy, followed closely by FWHM, 4SD and 5SD. Thresholding at 2SD and 3SD above remote signal intensity was less accurate, but there was still considerable overlap of confidence intervals with 6SD. Our data suggest that simple thresholding methods using the signal intensity of remote or of the enhanced area have relatively little effect on the assessment of regional viability and the prediction of likelihood of improvement. The lack of a clear difference may seem surprising considering the large differences that the use of the various methods caused in total infarct size and segmental extent of enhancement. However, with each method the inverse relation between SEE and likelihood of improvement remains intact, which makes it hard to detect small differences in predictive power. A larger study group and longer follow-up may be required to strengthen the statistical position of 6SD [3].

### Limitations

All current LGE quantification methods are limited by the fact that essential steps like the delineation of myocardial contours, the drawing a region of interest in remote myocardium and the exclusion of artifacts are all (still) done manually.

The applicability of our results is limited to patients with chronic ischemic heart disease, although they are probably valid in acute myocardial infarction, since both animal and clinical studies have shown that enhancement reflects necrosis at all stages after infarction [10-12]. However, this remains to be established. Also, our results cannot be extrapolated to other techniques (e.g. 3D-acquisition) or scanner types (especially with different magnetic field strengths).

In conclusion, in this study we evaluated the relation between simple thresholding techniques to quantify LGE images and functional outcome after revascularization in patients with chronic ischemic myocardial dysfunction. Although quantitative analysis with window setting thresholded at mean + 6SD of the signal of a remote normal region best predicted segmental functional improvement, there was no significant difference with the other SD or FWHM methods. Further study is needed to evaluate SD and FWHM in larger groups with a longer follow-up and compare them to the more complex algorithms that have not yet been tested in human viability studies.

## Competing interests

The authors declare that they have no competing interests.

## Authors' contributions

AMB participated in the design of the study, data acquisition, image analysis, statistical analysis and drafting of the manuscript. OB and FA participated in the data acquisition and the image analysis. ACvR participated in the design of the study and drafting of the manuscript. All authors have read and approved the manuscript.
